# Apparent diffusion coefficient for the prediction of tumor response to neoadjuvant chemo-radiotherapy in locally advanced rectal cancer

**DOI:** 10.1186/s13014-020-01738-6

**Published:** 2021-01-20

**Authors:** Mengjing Zhao, Lihao Zhao, Han Yang, Yuxia Duan, Gang Li

**Affiliations:** 1 Department of Radiology, The First Affiliated Hospital of Wenzhou Medical University, Wenzhou, Zhejiang People’s Republic of China; 2 Department of Chemoradiation Oncology, The First Affiliated Hospital of Wenzhou Medical University, Wenzhou, Zhejiang People’s Republic of China

**Keywords:** Apparent diffusion coefficient, Neoadjuvant chemo-radiotherapy, Locally advanced rectal cancer, Tumor response rate, Primary tumor progression-free survival

## Abstract

**Background:**

Patients with locally advanced rectal cancer generally have different response rates to preoperative neoadjuvant chemo-radiotherapy. This study investigated the value of the apparent diffusion coefficient (ADC) as a predictor to forecast the response to neoadjuvant chemo-radiotherapy in patients with locally advanced rectal cancer.

**Methods:**

Ninety-one locally advanced rectal cancer patients who underwent neoadjuvant chemo-radiotherapy between 2015 and 2018 were enrolled. Diffusion-weighted magnetic resonance imaging was performed before treatment and within 4 weeks after the completion of neoadjuvant chemo-radiotherapy. Mean ADC values of regions of interest were evaluated by two radiologists. The tumor response was evaluated according to RESCIST 1.1. The cut-off value for the mean ADC and increasing percentage (ΔADC%) after neoadjuvant chemo-radiotherapy was calculated using the receiver operating characteristic curve. The response rate of pre-ADC and ΔADC% above/below the cut-off values was determined using the chi-square test, respectively. Primary tumor progression-free survival (PFS) was analyzed using the Kaplan–Meier method, based on the pre-ADC and ΔADC% cut-off values.

**Results:**

The cut-off value of mean pre-ADC and ΔADC% was 0.94 × 10^–3^ mm^2^/s (80.36% sensitivity, 74.29% specificity) and 26.0% (73.21% sensitivity, 77.14% specificity), respectively. Lower mean pre-ADC values were related to a better response rate (83.3% vs 29.7%, *P* < 0.001) and PFS (26.12 vs 17.70 months, *P* = 0.004). ΔADC% above the cut-off value was also related to a better response rate (83.7% vs 35.7%, *P* < 0.001) and PFS (26.93 vs 15.65 months, *P* = 0.034).

**Conclusions:**

The mean ADC pre-treatment value and ΔADC% were potential predictors for the tumor response in locally advanced rectal cancer patients treated with neoadjuvant chemo-radiotherapy.

## Background

Colorectal cancer ranks as the third most common malignancy and remains the leading cause of cancer-related human mortalities worldwide [1]. Rectal cancer has a greater risk of local recurrence as resection is more difficult because of anatomical limitations. Colorectal cancer also has a higher risk of metastasizing to the lungs than colon cancer [2, 3]. At present, surgical resection is considered to be an effective treatment for rectal cancer [4]. However, for patients with lymph node metastasis or adjacent organs invasion, the prognosis is typically unsatisfactory [5]. Patients diagnosed at stage IV have a 5-year mortality of about 90% [6]. For patients with locally advanced lesions, neoadjuvant chemo-radiotherapy has been used to down-stage the primary tumor and offer the chance for radical operation [7, 8].

Magnetic resonance imaging (MRI) is often used to evaluate the status of rectal cancer. The precise anatomy of the rectum and adjacent organs can be shown via MRI, which can provide relatively accurate staging of primary tumors. Studies have reported that MRI can also predict the risk of local recurrence and distant metastasis [9]. However, the traditional MRI method is not available for accessing the response of the tumor to neoadjuvant chemo-radiotherapy. The measurement of tumor size after treatment is typically interfered with by necrosis and other factors. Diffusion-weighted imaging (DWI) is a distinct functional MRI technique that is sensitive to the movement of water molecules in vivo. Moreover, the apparent diffusion coefficient (ADC) has become a quantitative parameter used to evaluate the magnitude of the motion of the diffusion of water through tissue, showing information related to tissue cellularity [10, 11]. The movement caused by diffusion is mainly affected by the properties of the tissues and cells, the integrity of cell membranes, and the viscosity of fluids [12]. ADC values have been proven to correlate with tumor cellularity and grade [13]. As a potential predictor for the early prediction of therapeutic response, ADC parameters have been confirmed in various types of cancer, such as head and neck cancer, Hodgkin lymphoma, lung cancer, breast cancer, and prostate cancer [14–18].

Although the ADC value as a pre-treatment predictor is controversial, it remains a potential application prospect. In this study, the ADC was investigated as a predictor to forecast the response to neoadjuvant chemo-radiotherapy in patients with locally advanced rectal cancer and to provide insight into the treatment of locally advanced rectal cancer.

## Patients and methods

### Patients

One hundred and seventy-six locally advanced rectal cancer patients who underwent neoadjuvant chemo-radiotherapy in the First Affiliated Hospital of Wenzhou Medical University from September 2015 to May 2018 were reviewed. These patients were diagnosed with adenocarcinoma via endoscopic biopsy. Patients who underwent completed neoadjuvant chemo-radiotherapy and had available MRIs with the DWI series before and within 1 month after neoadjuvant chemo-radiotherapy started, were included.

Forty-seven patients who did not complete the entirety of the neoadjuvant chemo-radiotherapy were excluded. Thirty-eight patients did not have at least one MRI scan with the DWI series within 1 month since neoadjuvant chemo-radiotherapy started. Finally, 91 patients were enrolled in our study, all of the patients with locally advanced rectal cancer had at least one measurable target lesion in RECIST standard. The characteristics of the patients are described in Table 1. This study was performed at the First Affiliated Hospital of Wenzhou Medical University and approved by the Institutional Review Board. The requirement for written informed consent was waived.

**Table 1:** Clinical patients characteristics

Variables	Values	Range or percent
Total no. of patients	91	
Age, years (median)	61	38–82
< 70	78	85.7%
≥ 70	13	14.3%
Gender		
Male	61	67.0%
Female	30	33.0%
Clinical tumor depth		
T2	4	4.2%
T3	85	93.4%
T4	2	2.2%
Clinical lymph node metastases		
N0	41	45.0%
N1	28	30.8%
N2	22	24.2%
Histological classification		
High differentiation	11	12.1%
Moderate differentiation	76	83.5%
Low differentiation	4	4.4%
Treatment response		
CR	0	0.0%
PR	56	61.5%
SD	28	30.8%
PD	7	7.7%
Post-operation pathological response		
Response (pCR + pPR)	20	22.0%
Non-response	71	78.0%

*CR* complete response, *PR* partial response, *SD* stable disease, *PD* progressive disease, *pCR* pathological complete response, *pPR* pathological partial response

### MR imaging

All patients underwent both a pre-treatment MR imaging examination for primary tumors and a second MR imaging, after the completion of neoadjuvant chemo-radiotherapy treatment, and 1–4 weeks before the operation. All subjects underwent two MRI examinations using a 3.0-T MR scanner (Signa HDX, General Electric, Ltd) equipped with a phased array body coil. Before the MRI examinations, all patients did shallow and slow uniform breathing exercises to collect the required signals. All 91 patients underwent a series of MRI scans, including T1WI, T2WI, T2WI short-tau inversion recovery (STIR) contrast-enhanced T1WI, FLAIR, and DWI. All MRI examinations contained T1 weighted imaging (TR/TE 600/6 ms, average number 1, FOV 350 × 350 mm, matrix 256, slice thickness 4 mm, skip 1.2–1.6 mm, and slice 20) and T2 weighted imaging (TR/TE 3460/105 ms, concatenations 2, flip angle 180°, matrix 320, average number 2, FOV 240 × 240 mm, slice thickness 4 mm, slice 20, and skip 1.2–1.6 mm). DWI scans were obtained using a single-shot spin-echo type of echo-planar sequence, and fat signals were suppressed using STIR. The b-values of DWI were b = 0 and 1000 s/mm^2^.

### Imaging analysis, regions of interest (ROI), and assessment of response

Both MR images were transferred into a workstation (Philips Medical Systems, The Netherlands). According to the images obtained from DWI, the corresponding ADC map was obtained by DWI image fusion, automatically, with Functool software 9.4.04b (ADW 4.5, General Electric, Ltd) when the b-value was 0 and 1000 s/mm^2^. Pre- and post-treatment DWI images were analyzed to define the tumor, with the tumor being defined as high signal intensity corresponding to the location of the tumor mass on the DWI images. In general, DWI images have a higher resolution than ADC maps. ROIs were manually placed on DWI images with a b-value of 1000 s/mm^2^, and the ROIs were copied to the corresponding ADC maps. Pre- and post-treatment MR images were compared to ensure that ROIs were placed within the location of the primary tumor. In several patients, high signal intensity zones were not discovered on post-treatment neoadjuvant chemo-radiotherapy DWI images, and ROIs were placed in the before neoadjuvant chemo-radiotherapy treatment tumor location. The areas of the ROIs were positioned on a single trans-axial slice where the section containing the largest tumor area was avoiding areas of fibrosis and necrosis, and where the mean ADC value or ROI was located. Two radiologists independently analyzed pre- and post-treatment MR and DW images and performed the ADC measurements. The radiologists were blinded to the pathology reports, clinical patient data, and each other’s results. In MRI before and after neoadjuvant chemo-radiotherapy treatment, the ROIs were drawn on the same anatomical lesion. The mean ADC value of a single ROI calculated by the two radiologists was averaged as an observation for analysis and labeled as the ADC of the tumor. The pre-treatment and post-treatment ADCs were labeled as pre-ADC and post-ADC, respectively, and ΔADC% was calculated according to the following equation: (post-ADC − pre-ADC)/pre-ADC × 100. The treatment response was accessed according to RESCIST 1.1 by the same radiologists, to reach an agreement. Primary tumor complete response (CR) and Primary tumor partial response (PR) were defined as ‘response,’ and Primary tumor stable disease (SD) and Primary tumor progressive disease (PD) were defined as ‘no-response.’

### Preoperative treatment

All patients were treated with long-term chemo-radiotherapy that included the following: Radiotherapy applied with the 3D-conformational multiple field technique. All treatment volumes and organs at risk (OAR) contouring were reviewed. The gross tumor volume (GTV) was defined using MRI, including the rectal tumor and its corresponding mesorectum region. The clinical target volume (CTV) included mesorectum region, superior rectal artery lymph nodes, internal iliac lymph nodes, obturator lymph nodes, presacral lymph nodes, external iliac lymph nodes (selective delineation at T4b) and inguinal lymph nodes drainage area (when lymph node metastasis was confirmed). The prescribed dose was 50 Gy in 25 fractions to GTV and 45 Gy in 25 fractions to CTV in plans for 5 weeks. Preoperative chemotherapy was delivered in two 5-day courses during the first and fifth weeks of radiotherapy. Fluorouracil was given as a 120-h continuous injection at a dose of 1000 mg per square meter per day.

### Statistical analysis

The primary aim of this study was to ascertain whether the single-plane mean ADC value of the ROI before neoadjuvant chemo-radiotherapy treatment could be a predictor of response. At the primary analysis step, Interobserver agreement of ADC value measurements (performed ADC of ROI before and after treatment of neoadjuvant chemo-radiotherapy) was analyzed using the Pearson correlation test. The mean pre- and post-treatment ADC values were compared using the Mann–Whitney U test for the ROI positioning method. The mean ADC values and difference values of the change in ADC obtained by the ROI positioning method were compared using the nonparametric Wilcoxon rank-sum test with regard to the group of patients with tumor response (including the CR and PR groups) and the group of patients with noncomplete tumor response (including SD and PD group). Receiver operating characteristic (ROC) curves were generated to detect an eligible cut-off value of the mean ADC values pre-treatment of neoadjuvant chemo-radiotherapy. All patients were divided into two groups according to the mean ADC pre-treatment neoadjuvant chemo-radiotherapy (above/below the cut-off value), and the chi-square test was conducted to find the difference in the response rate between the two groups. Primary tumor progression-free survival (PFS) was calculated using the log-rank test stratified by the mean ADC pre-treatment of neoadjuvant chemo-radiotherapy and the ΔADC% after neoadjuvant chemo-radiotherapy. All results were plotted using the Kaplan–Meier method. All statistical analyses and graphs in this article were performed in commercially available statistical software (version 19.0, SPSS Statistics Software, Inc.) and graphic software (version 8.02, Prism, Inc.), respectively. *P* values less than 0.05 were considered to indicate a statistically significant difference.

## Results

There was a good interobserver agreement between the two radiologists regarding the average single-plane pre-ADC, post-ADC, and ΔADC values (0.851, 0.824, 0.867, respectively; *P* < 0.001 for each one).

### Response rate in total patients

Within 1 month after the neoadjuvant chemo-radiotherapy, 56 (61.54%) patients obtained PR, 28 (30.77%) patients developed SD, and 7 (7.69%) obtained PD. There were no patients who obtained CR. The response (CR + PR) and no-response (SD + PD) rates were 61.54% and 38.46%, respectively. A total of 20 (21.98%) patients had complete and partial pathological response of tumors. There were 71 (78.02%) patients with non-response in pathologically. ΔADC% was a significant factor associated with pathological response of tumor to neoadjuvant chemo-radiotherapy (*P* = 0.033). The results of the ADC measurement and patient characteristics in the multivariable linear regression analyses are described in Table 2. The pre-ADC and ΔADC% were two significant factors associated with primary tumor response to neoadjuvant chemo-radiotherapy.

**Table 2:** Comparison of characteristics between patients with response and those without response

Parameter	OR	95% CI	*P* value
Univariate analysis			
Age (years)	1.015	0.976–1.057	0.451
Male sex	1.284	0.512–3.217	0.594
Clinical tumor depth			
T2	1	–	0.814
T3	3	0.084–107.447	0.547
T4	1.576	0.095–26.067	0.751
Clinical lymph node metastases			
N0	1	–	0.189
N1	2.160	0.756–6.173	0.151
N2	2.850	0.878–9.252	0.081
Histological classification			
High differentiation	1	–	0.310
Moderate differentiation	3.600	0.280–46.359	0.326
Low differentiation	5.444	0.540–54.927	0.151
Pre-ADC	11.818	4.328–32.272	< 0.001
Post-ADC	2.312	0.974–5.492	0.058
ΔADC%	9.225	3.441–24.728	< 0.001
Multivariable analysis			
Clinical Lymph node metastases			
N0	1	–	0.129
N1	6.226	0.253–153.237	0.263
N2	15.486	0.871–275.198	0.062
Pre-ADC	5.521	1.344–22.673	0.018
Post-ADC	2.108	0.433–10.259	0.355
ΔADC%	9.886	2.460–39.720	0.001

### Mean ADC values in ROI before treatment

**Fig. 1:**
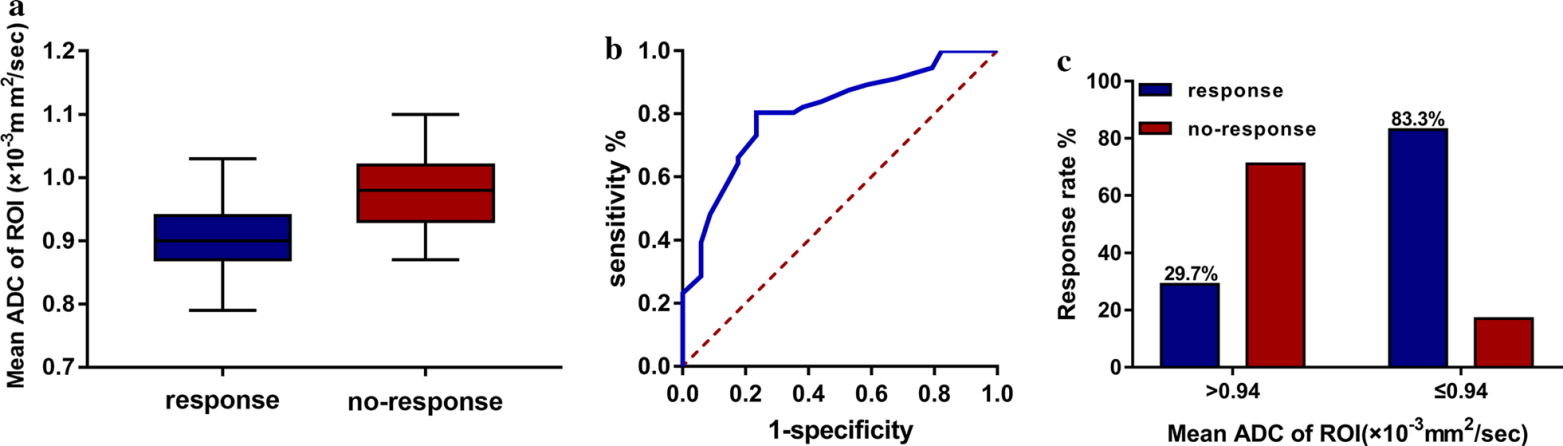
Mean ADC value of ROI pre-treatment. **a** Mean pre-ADC value of ROI with different primary tumor response; **b** ROC cure of mean pre-ADC value of ROI to predict primary tumor response; **c** Primary tumor response rate with mean pre-ADC value of ROI above or below a cut-off value (0.94 × 10^−3^ mm^2^/s)

The tumor mean ADC values of ROI pre-treatment were significantly lower in patients with good response than patients with no response (*P* < 0.001, Fig. 1a). The predict performance of the mean ADC value of ROI pre-treatment in the prediction of primary tumor response was evaluated by ROC curve analysis (Fig. 1b). The optimal cut-off value for the pre-ADC was 0.94 × 10^–3^ mm^2^/s, and the area under the ROC curve was 0.801 (95% confidence interval, 0.704–0.877). The tumor ADC appropriate sensitivity and specificity were 80.36% and 74.29%, respectively. The primary tumor response rate was significantly higher in patients with a mean ADC value of ROI below 0.94 × 10^–3^ mm^2^/s (83.3%) than that above it (29.7%) (*P* < 0.001, Fig. 1c).

### Mean ADC values of ROI after treatment

**Fig. 2:**
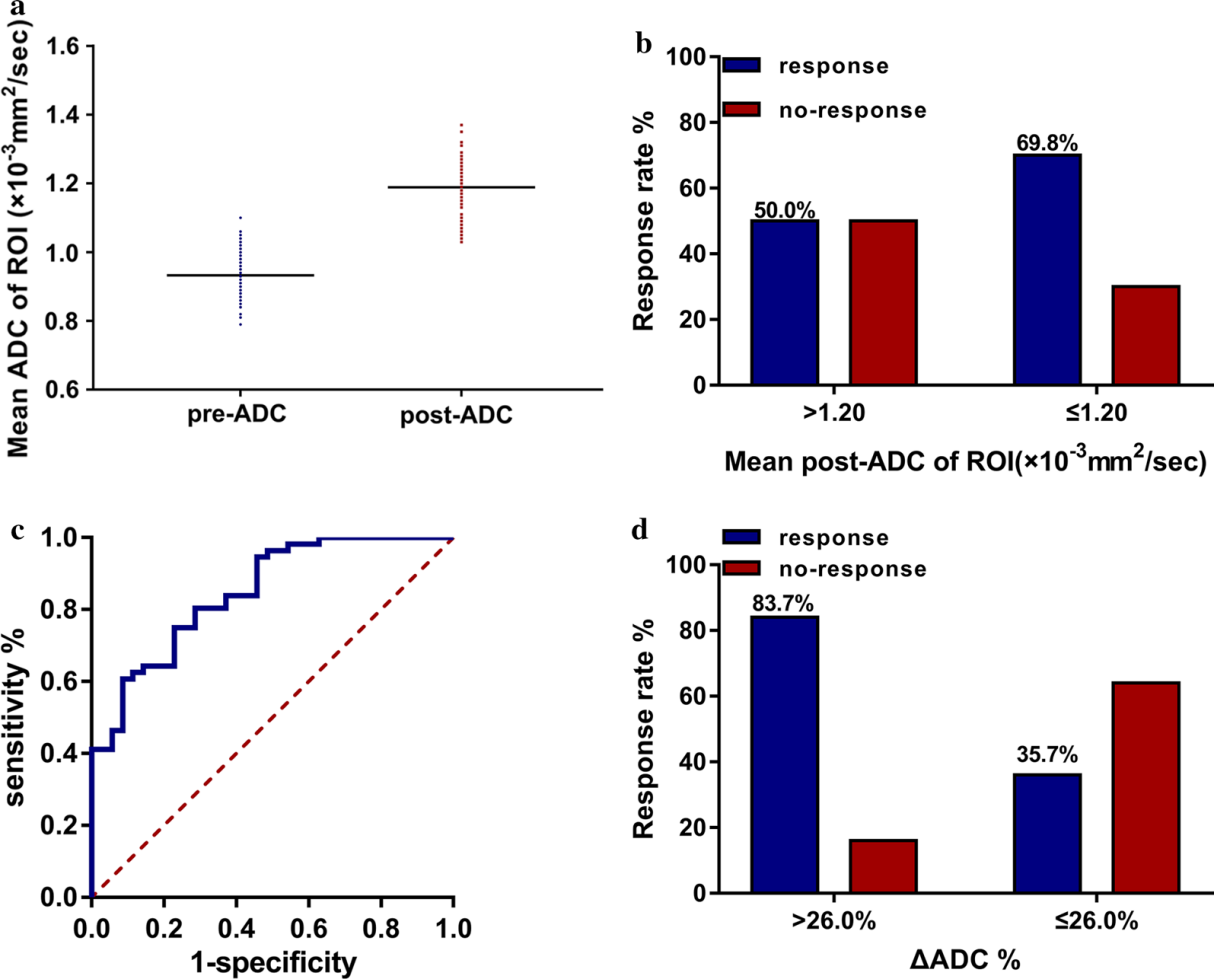
Mean ADC value of ROI post-treatment. **a** Mean pre-ADC and post-ADC; **b **ΔADC with different response after neoadjuvant chemoradiotherapy; **c** ROC cure of ΔADC% post-neoadjuvant chemoradiotherapy to predict primary tumor response; **d** primary tumor response rate with ΔADC% above or below a cut-off value (26.0%)

**Fig. 3:**
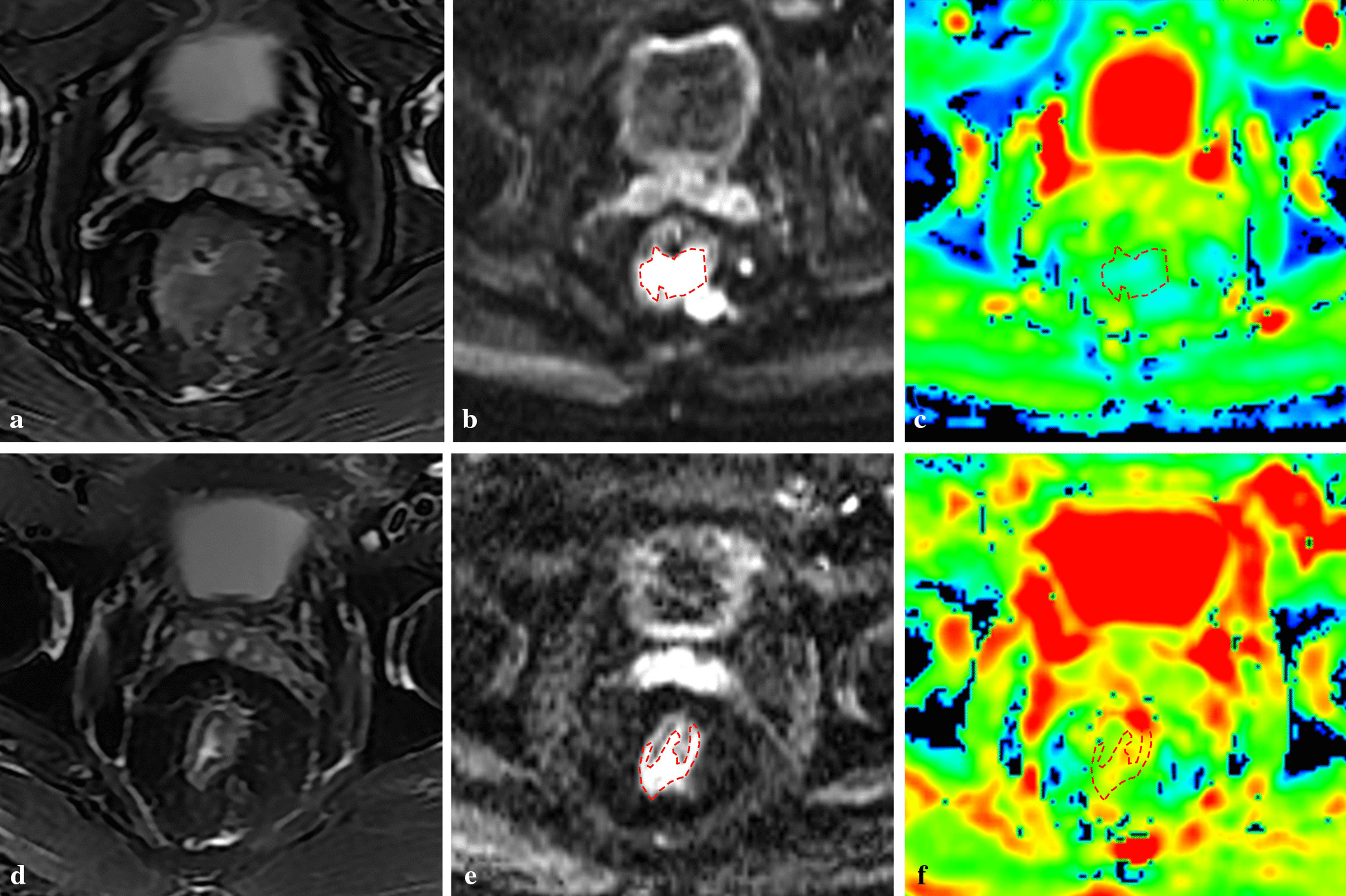
MRI change of rectal cancer before and after treatment of neoadjuvant chemo-radiotherapy. **a** T2WI STIR before treatment of neoadjuvant chemoradiotherapy; **b** DWI before treatment of neoadjuvant chemoradiotherapy; **c** ADC map before treatment of neoadjuvant chemoradiotherapy; **d** T2WI STIR after treatment of neoadjuvant chemoradiotherapy; **e** DWI after treatment of neoadjuvant chemoradiotherapy; **f** ADC map after treatment of neoadjuvant chemoradiotherapy;

The tumor mean ADC values of ROI significantly increased after treatment within 1 month (*P* = 0.001 Figs. 2a, 3). The predict performance of the mean ADC value of ROI post-treatment was determined by ROC curve analysis. The optimal cut-off value for the post-ADC was 1.20 × 10^–3^ mm^2^/s, and the area under the ROC curve was 0.567 (*P* = 0.284). The tumor ADC appropriate sensitivity and specificity were 66.07% and 54.29%, respectively. The primary tumor response rate was slightly higher in patients with a mean ADC value of ROI below 1.20 × 10^–3^ mm^2^/s (69.8%) than that above it (50.0%) (*P* = 0.057, Fig. 2b). The patients with a good primary tumor response had a higher increased percentage ΔADC value (ΔADC %) compared to those with a poor response (*P* < 0.001). Using ROC curve analysis, a cut-off value to predict tumor response after treatment was based on the percentage ΔADC value (ΔADC %) (Fig. 2c). The optimal cut-off value for ΔADC% was 26%, and the area under the ROC curve was 0.848. We obtained an appropriate sensitivity of 73.21% and a specificity of 77.14%. Patients achieved a relatively higher ΔADC%, which indicated a better tumor response to treatment (83.7% vs. 35.7%, *P* < 0.001, Fig. 2d).

### The primary tumor progression-free survival

**Fig. 4:**
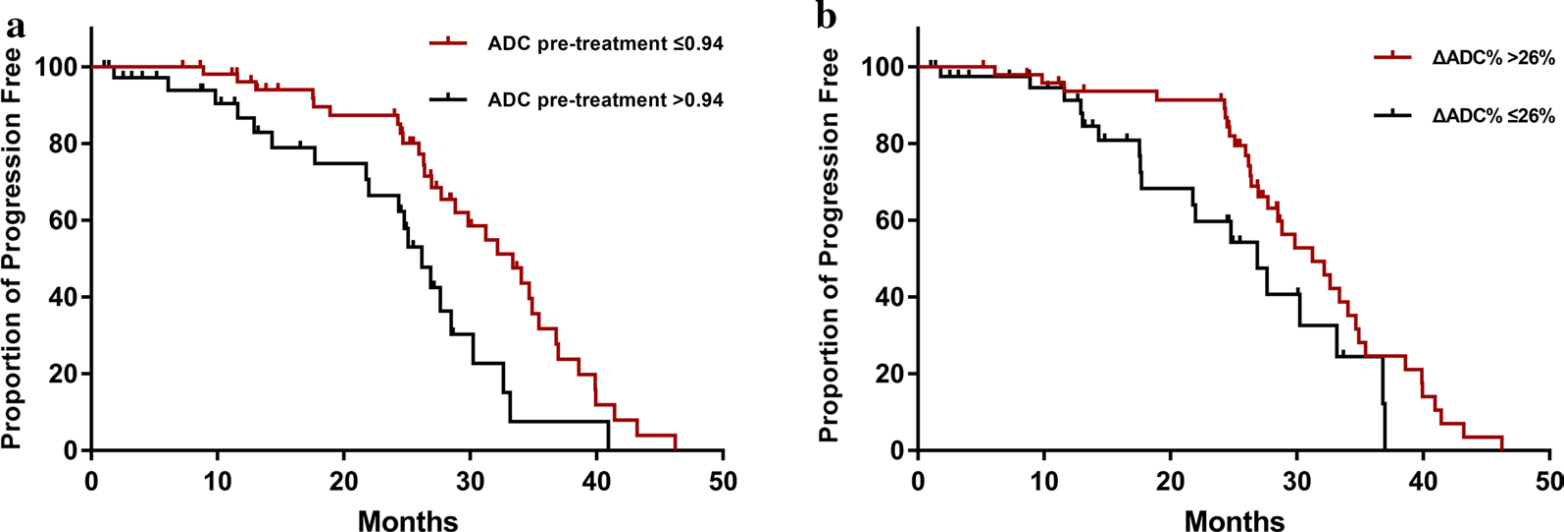
Primary tumor progression free survival (PFS). **a** PFS of Mean pre-ADC value of ROI divided with cut off value 0.94 × 10^−3^ mm^2^/s; **b** PFS of ΔADC% divided with cut off value 26.0%

With a median follow-up of 24.67 (IQR 13.08, 28.73) months, the 2-years progression-free survival rate was 60.44%. The median PFS of the good response group and no-response group was 26.62 (IQR 19.62, 33.07) months and 14.30 (IQR 8.67, 24.93) months, respectively. The relationship between the mean ADC values of ROI pre-treatment (*P* = 0.004) and the ΔADC% post-treatment (*P* = 0.034) with PFS reached statistical significance based on the cut-off value (Fig. 4).

## Discussion

The results of this study demonstrated that the mean ADC value of ROI and ΔADC% could be used as a predictor to predict the response to neoadjuvant chemo-radiotherapy in rectal cancer patients. A low pre-treatment mean tumor ADC appears to predict a good response to neoadjuvant chemo-radiotherapy; likewise, the ΔADC% post-treatment is related to tumor response. Overall, the results illustrate that the ADC appears to be a promising tool for facilitating predictions and monitoring the response to neoadjuvant chemo-radiotherapy for rectal cancer patients.

Preoperative chemo-radiotherapy for patients with resectable rectal cancer is based on the expected survival benefit achieved with this treatment [19]. These advantages are that the chemo-radiotherapy regimen is carried out before major surgery and the compliance of patients can be improved, as well as down-staging, which may enhance the surgical cure rate and permit sphincter preservation with low-lying tumors [20]. In addition, because tumor oxygenation is better with preoperative treatment than with postoperative treatment, irradiation seems to be more effective with the preoperative treatment approach [21]. It has been reported that preoperative administration of chemo-radiotherapy significantly prolonged disease-free survival compared with postoperative administration and demonstrated a trend toward improved overall survival (OS) [22]. However, there are significant differences in individual response to preoperative chemo-radiotherapy and ineffective treatment, which may result in unnecessary toxicity to the patients as well as the delay of appropriate treatment [23]. Generally, several weeks should be taken to confirm the treatment response after neoadjuvant chemo-radiotherapy. This delay may cause some patients to miss the optimal time for surgical treatment, causing negative impacts on patients’ survival and recovery. An effective and simple early prediction of treatment response to neoadjuvant chemo-radiotherapy will provide an opportunity for rectal cancer patients to find early treatment response and improve the treatment effect. At the primary analysis step, we obtained the results in the multivariable linear regression analyses and found that the pre-ADC and ΔADC% were two significant factors associated with primary tumor response to neoadjuvant chemo-radiotherapy. Moreover, we focused on the application of the ADC value in advanced rectal cancer treated by neoadjuvant chemo-radiotherapy. As far as we know, there is currently controversy surrounding this topic.

Diffusion-weighted MRI (DW-MRI) as a noninvasive imaging technique based on the Brownian movement of water molecules, has been used in the diagnosis of early rectal cancer [24]. The DWI-derived ADC is a quantitative parameter that can be used to evaluate properties of molecules, including cell density, cell membrane integrity, and interactions with macromolecules. Generally, ADC values are negatively related to cell density and positively related to extracellular space [25]. It has been proven that the ADC can reflect histological changes in primary tumors after neoadjuvant chemo-radiotherapy [26]. Several investigators studying rectal cancer have reported that the pre-treatment mean ADC value of the responder group had a statistically lower ADC than that of the non-responder group [21, 27, 28]. We obtained results similar to those reported, as primary rectal tumors with lower pre-chemo-radiotherapy ADC values were more likely to have a better response after treatment. Low ADC values before treatment indicate that the cellular density of the primary tumor is lower, leading to better cellular oxygenation and causing increased sensitivity to radiation. However, it should be noted that our results showing post-ADC values did not reach statistical significance related to primary rectal tumor response to neoadjuvant chemo-radiotherapy. In this study, a higher ΔADC% post-neoadjuvant chemo-radiotherapy was related to better primary tumor response. The ΔADC values performed well in discriminating noncomplete responders eligible for surgery [29]. The increase in ADC values may be related to a decrease in tumor cell density. Also noteworthy was the higher diagnostic performance of the percentage of ADC change than volume change detected by traditional methods [29]. Thus, the quantitative analysis of ADC can serve as a sensitive predictor for detecting the efficacy of neoadjuvant chemo-radiotherapy for rectal cancer. In our study, the mean ADC value and ΔADC% were statistically significantly related to a better PFS. Moreover, the lower mean ADC group and higher ΔADC% group predict patients had a prolonged PFS trend.

There are several limitations to the current study. First, our study conducted with relatively a small number of patients of a single center, and more data is needed to further estimate the prediction of ADC. Second, several results indicated that variations in ROI number, size, and position, influence tumor ADC measurements; we selected only one image slice within the tumor, which may have the potential of ignoring tumor heterogeneity, and addressed it as a study limitation. This common statistic has the merit of simplicity. Finally, we were unable to evaluate interobserver variability in ADC measurements because we attempted to achieve more consistent ADC measurements by an experienced reader.

## Conclusions

ADC measurements induced by neoadjuvant chemo-radiotherapy may have considerable diagnostic value for the estimation of complete tumor response. ADC values are effective and convenient potential predictors for predicting the tumor response in rectal cancer patients, compared with traditional imaging evaluation methods. The change in ADC value also demonstrated excellent performance in distinguishing treatment response, which can help oncologists improve their therapy plans, avoid missing opportunities for surgical treatment, and avoid unnecessary toxicity to patients.

## Data Availability

The datasets used and analyzed during the current study are available from the corresponding author on reasonable request.

## Abbreviations

ADC: Apparent diffusion coefficient
AUC: Area under the curve
DWI: Diffusion-weighted imaging
CR: Complete response
PD: Progressive disease
PR: Partial response
PFS: Progression free survival
MRI: Magnetic resonance imaging
RECIST: Response Evaluation Criteria in Solid Tumors
ROI: Region of interest
ROC: Receiver-Operating-Characteristic
T1WI: T1 weighted image
T2WI: T2 weighted image
TE/TR: Echo time/repetition time

## References

[1] Ferlay J, Colombet M, Soerjomataram I, Mathers C, Parkin DM, Pineros M, Znaor A, Bray F (2019). Estimating the global cancer incidence and mortality in 2018: GLOBOCAN sources and methods. Int J Cancer.

[2] Hong TS, Clark JW, Haigis KM (2012). Cancers of the colon and rectum: identical or fraternal twins?. Cancer Discov.

[3] Bosset JF, Collette L, Calais G, Mineur L, Maingon P, Radosevic-Jelic L, Daban A, Bardet E, Beny A, Ollier JC (2006). Chemotherapy with preoperative radiotherapy in rectal cancer. N Engl J Med.

[4] Wu CC, Lee RC, Chang CY (2013). Prediction of lymphovascular invasion in rectal cancer by preoperative CT. AJR Am J Roentgenol.

[5] Liu T, Li C, Jin L, Li C, Wang L (2019). The prognostic value of m6A RNA methylation regulators in colon adenocarcinoma. Med Sci Monit.

[6] Lin JS, Piper MA, Perdue LA, Rutter C, Webber EM, O'Connor E, Smith N, Whitlock EP. In: Screening for colorectal cancer: a systematic review for the US preventive services task force*.* edn. Rockville (MD); 2016.

[7] Chen EY, Kardosh A, Nabavizadeh N, Lopez CD (2019). Evolving treatment options and future directions for locally advanced rectal cancer. Clin Colorectal Cancer.

[8] Dighe S, Swift I, Magill L, Handley K, Gray R, Quirke P, Morton D, Seymour M, Warren B, Brown G (2012). Accuracy of radiological staging in identifying high-risk colon cancer patients suitable for neoadjuvant chemotherapy: a multicentre experience. Colorectal Dis.

[9] Glynne-Jones R, Wyrwicz L, Tiret E, Brown G, Rodel C, Cervantes A, Arnold D, Committee EG (2018). Rectal cancer: ESMO clinical practice guidelines for diagnosis, treatment and follow-up. Ann Oncol.

[10] Sun YQ, Tong T, Cai SJ, Bi R, Xin C, Gu YJ (2014). Apparent diffusion coefficient (ADC) value: a potential imaging biomarker that reflects the biological features of rectal cancer. PLoS ONE.

[11] Metcalfe P, Liney GP, Holloway L, Walker A, Barton M, Delaney GP, Vinod S, Tome W (2013). The potential for an enhanced role for MRI in radiation-therapy treatment planning. Technol Cancer Res Treat.

[12] Akashi M, Nakahusa Y, Yakabe T, Egashira Y, Koga Y, Sumi K, Noshiro H, Irie H, Tokunaga O, Miyazaki K (2014). Assessment of aggressiveness of rectal cancer using 3-T MRI: correlation between the apparent diffusion coefficient as a potential imaging biomarker and histologic prognostic factors. Acta Radiol.

[13] Barbaro B, Vitale R, Valentini V, Illuminati S, Vecchio FM, Rizzo G, Gambacorta MA, Coco C, Crucitti A, Persiani R (2012). Diffusion-weighted magnetic resonance imaging in monitoring rectal cancer response to neoadjuvant chemoradiotherapy. Int J Radiat Oncol.

[14] Martins EB, Chojniak R, Kowalski LP, Nicolau UR, Lima EN, Bitencourt AG (2015). Diffusion-weighted MRI in the assessment of early treatment response in patients with squamous-cell carcinoma of the head and neck: comparison with morphological and PET/CT findings. PLoS ONE.

[15] Punwani S, Taylor SA, Saad ZZ, Bainbridge A, Groves A, Daw S, Shankar A, Halligan S, Humphries PD (2013). Diffusion-weighted MRI of lymphoma: prognostic utility and implications for PET/MRI?. Eur J Nucl Med Mol Imaging.

[16] Shintani T, Matsuo Y, Iizuka Y, Mitsuyoshi T, Umeoka S, Nakamoto Y, Mizowaki T, Togashi K, Hiraoka M (2017). Assessment of treatment response after lung stereotactic body radiotherapy using diffusion weighted magnetic resonance imaging and positron emission tomography: a pilot study. Eur J Radiol.

[17] Sampath S, Rahmanuddin S, Sahoo P, Frankel P, Boswell S, Wong J, Rotter A, Rockne R, Wong J, Park JM (2019). Change in apparent diffusion coefficient is associated with local failure after stereotactic body radiation therapy for non-small cell lung cancer: a prospective clinical trial. Int J Radiat Oncol.

[18] Barnes SL, Sorace AG, Whisenant JG, McIntyre JO, Kang H, Yankeelov TE (2017). DCE- and DW-MRI as early imaging biomarkers of treatment response in a preclinical model of triple negative breast cancer. NMR Biomed.

[19] Sauer R, Becker H, Hohenberger W, Rodel C, Wittekind C, Fietkau R, Martus P, Tschmelitsch J, Hager E, Hess CF (2004). Preoperative versus postoperative chemoradiotherapy for rectal cancer. N Engl J Med.

[20] Colorectal Cancer Collaborative G (2001). Adjuvant radiotherapy for rectal cancer: a systematic overview of 8,507 patients from 22 randomised trials. Lancet.

[21] Dzik-Jurasz A, Domenig C, George M, Wolber J, Padhani A, Brown G, Doran S (2002). Diffusion MRI for prediction of response of rectal cancer to chemoradiation. Lancet.

[22] Roh MS, Colangelo LH, O'Connell MJ, Yothers G, Deutsch M, Allegra CJ, Kahlenberg MS, Baez-Diaz L, Ursiny CS, Petrelli NJ (2009). Preoperative multimodality therapy improves disease-free survival in patients with carcinoma of the rectum: NSABP R-03. J Clin Oncol.

[23] Patterson DM, Padhani AR, Collins DJ (2008). Technology insight: water diffusion MRI–a potential new biomarker of response to cancer therapy. Nat Clin Pract Oncol.

[24] Birlik B, Obuz F, Elibol FD, Celik AO, Sokmen S, Terzi C, Sagol O, Sarioglu S, Gorken I, Oztop I (2015). Diffusion-weighted MRI and MR- volumetry–in the evaluation of tumor response after preoperative chemoradiotherapy in patients with locally advanced rectal cancer. Magn Reson Imaging.

[25] Montelius M, Jalnefjord O, Spetz J, Nilsson O, Forssell-Aronsson E, Ljungberg M (2019). Multiparametric MR for non-invasive evaluation of tumour tissue histological characteristics after radionuclide therapy. NMR Biomed.

[26] Lin CC, Ou HY, Chuang YH, Chiang HJ, Yu CC, Lazo M, Tsang LL, Huang TL, Lin CC, Chen CL (2018). Diffusion-weighted magnetic resonance imaging in liver graft rejection. Transplant Proc.

[27] Sun YS, Zhang XP, Tang L, Ji JF, Gu J, Cai Y, Zhang XY (2010). Locally advanced rectal carcinoma treated with preoperative chemotherapy and radiation therapy: preliminary analysis of diffusion-weighted MR imaging for early detection of tumor histopathologic downstaging. Radiology.

[28] Hein PA, Kremser C, Judmaier W, Griebel J, Rudisch A, Pfeiffer KP, Hug EB, Lukas P, DeVries AF (2003). Diffusion-weighted MRI–a new parameter for advanced rectal carcinoma?. Rofo.

[29] Blazic IM, Lilic GB, Gajic MM (2017). Quantitative assessment of rectal cancer response to neoadjuvant combined chemotherapy and radiation therapy: comparison of three methods of positioning region of interest for ADC measurements at diffusion-weighted MR imaging. Radiology.

